# Enantiopurity by Directed Evolution of Crystal Stabilities and Nonequilibrium Crystallization

**DOI:** 10.1021/jacs.5c00569

**Published:** 2025-02-25

**Authors:** Clément Pinetre, Sjoerd W. van Dongen, Clément Brandel, Anne-Sophie Léonard, Maxime D. Charpentier, Valérie Dupray, Kasper Oosterling, Bernard Kaptein, Michel Leeman, Richard M. Kellogg, Joop H. ter Horst, Willem L. Noorduin

**Affiliations:** a Univ Rouen Normandie, Normandie Univ, SMS, UR 3233, Rouen F-76000, France; b AMOLF, Science Park 104, Amsterdam 1098 XG, The Netherlands; c EPSRC Future Continuous Manufacturing and Advanced Crystallisation Research Hub, c/o Strathclyde Institute of Pharmacy and Biomedical Sciences, University of Strathclyde, Glasgow G1 1RD, U.K.; d Symeres, Kadijk 3, Groningen 9747 AT, The Netherlands; e InnoSyn, Urmonderbaan 22, Geleen 6167 RD, The Netherlands; f Kellogg Beheer B.V., Zernikepark 12, Unit 1.31, Groningen 9747 AN, The Netherlands; g Tiofarma, Hermanus Boerhaavestraat 1, Oud-Beijerland 3261 ME, The Netherlands; h Van‘t Hoff Institute for Molecular Sciences, University of Amsterdam, Science Park 904, Amsterdam 1090 GD, The Netherlands

## Abstract

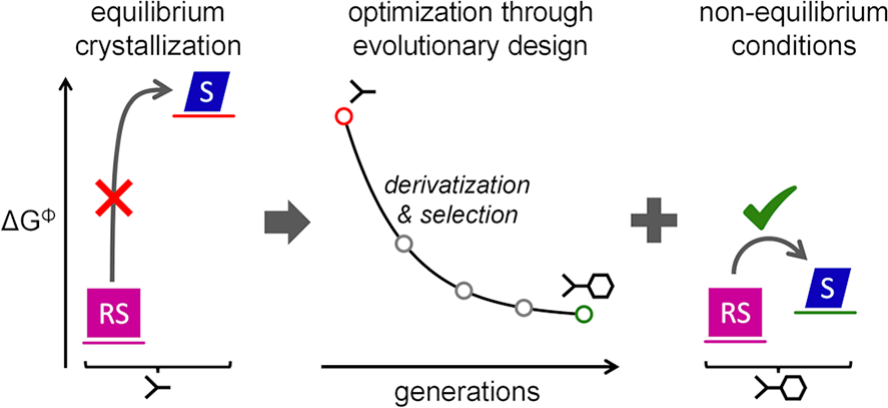

Crystallization is a powerful method to isolate enantiopure molecules from racemates if enantiomers self-sort into separate enantiopure crystals. Unfortunately, this behavior is unpredictable and rare (5–10%), as both enantiomers predominantly crystallize together to form racemic crystals, hindering any such chiral sorting. These unfavorable statistics might be overcome using nonequilibrium conditions. Therefore, we systematically characterize energy differences (Δ*G*^Φ^) between racemic and enantiopure crystal phases for libraries of target molecules (phenylglycine, praziquantel) with different chemical modifications. Surprisingly, these libraries reveal wide but similar continuous distributions of Δ*G*^Φ^, wherein similar chemical modifications group together. This grouping allows a directed evolution strategy to discover racemic crystals with low Δ*G*^Φ^ for isolating desired enantiomers by crystallization under nonequilibrium conditions. Comparison with over a hundred previously reported compounds suggests that as many as half of all chiral molecules may kinetically form enantiopure crystals (∼50%). These insights open new previously unconsidered possibilities for isolating enantiopure molecules.

## Introduction

**Figure 1:**
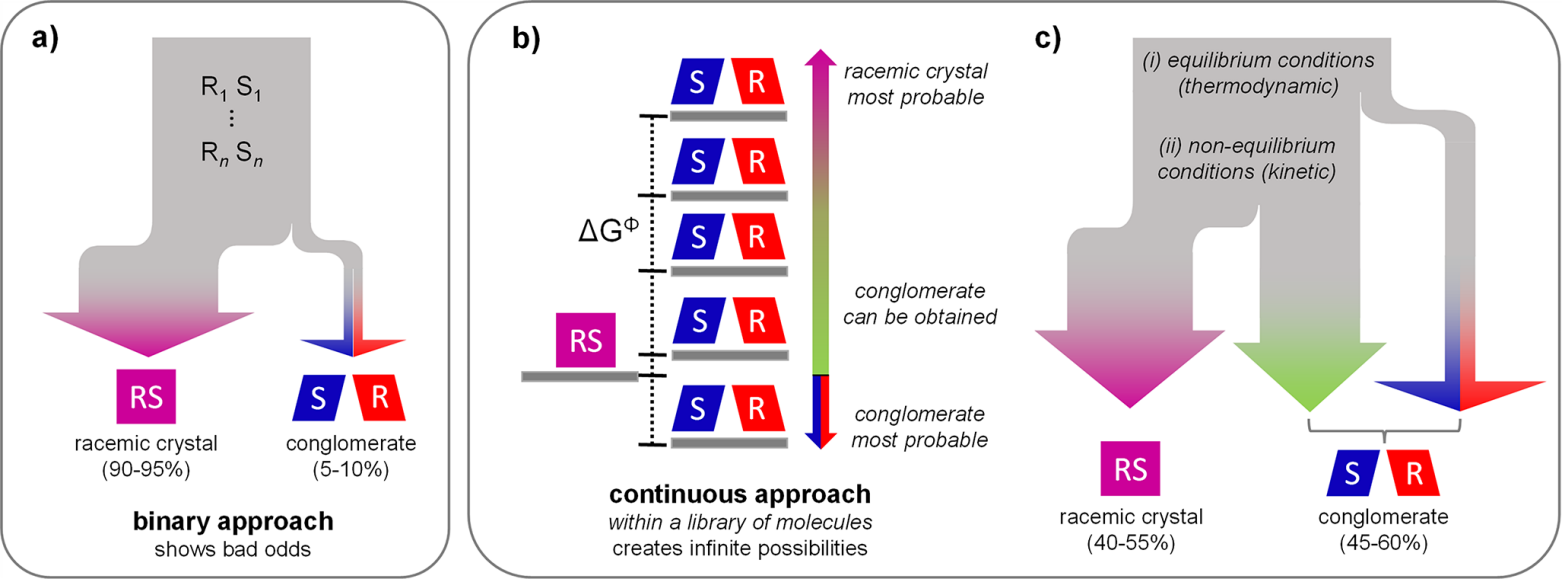
Stability of racemic compounds and conglomerates under thermodynamic and kinetic conditions. (a) Current binary approach: thermodynamics dictates whether chiral molecules form either racemic (90–95%) or conglomerate (5–10%) crystals. (b) Derivatives of a common chiral center yield a library that screens a continuous energy difference (Δ*G*^Φ^) between racemic and enantiopure crystal forms. (c) For small positive Δ*G*^Φ^, nonequilibrium conditions can (kinetically) stabilize enantiopure crystals of a thermodynamically stable racemic compound, such that an estimated 45–60% of chiral molecules may be accessible as either kinetic or thermodynamic conglomerates.

Crystallization is a simple, direct, and therefore common method to separate chiral molecules and isolate their pure enantiomers.^1−7^ However, chiral purification by crystallization has one fundamental requirement: enantiomers must spontaneously sort into separate enantiopure crystals (i.e., racemic conglomerates) (Figure 1a).^8^ Unfortunately, such self-sorting behavior is rare and unpredictable:^9,10^ the overwhelming majority of enantiomeric mixtures crystallize together into thermodynamically favored racemic compounds (90–95%), which complicates the use of direct crystallization for chiral separations.^11,12^ Overcoming the fundamental underlying thermodynamic limitations would not only open novel systematic and general approaches for discovering and utilizing conglomerates but also potentially allows the development of new strategies to resolve directly or even deracemize racemic compounds.

We here suggest that these thermodynamic limitations may be overcome by exploiting nonequilibrium conditions to kinetically favor the formation of conglomerates. Under such conditions, nucleation and crystal growth rates, rather than thermodynamic stabilities, may determine which crystalline phase dominates, akin to phenomena in polymorphism.^13,14^ This opens the potential to exploit nonequilibrium conditions for favoring kinetic conglomerates at the cost of thermodynamically stable racemic compounds. Supporting this idea, there have already been reports of racemic compounds converting into enantiopure crystals under far-from-equilibrium conditions by grinding crystals or by applying steep temperature gradients during cooling crystallization.^15−20^ Although promising, it remains unclear if such cases are incidental reports on systems with specific traits or if there are general guidelines that can be exploited to extend these principles for the systematic isolation of enantiomers by crystallization.

The conversion from racemic crystal phases into their enantiopure crystal counterparts may be feasible when the energy difference Δ*G*^Φ^ between both phases is small (Figure 1b).^21^ Indeed, for polymorphic transformations, it is commonly accepted that when Δ*G*^Φ^ < 0.5 kcal mol^–1^ (2.1 kJ mol^–1^) thermodynamically stable phases may be converted into kinetically stable crystal phases.^22−24^ Previously, Δ*G*^Φ^ has been analyzed for many chiral compounds and has been used as an indicator for identifying thermodynamically stable racemic conglomerates.^25−27^ However, these earlier analyses concerned incidental reports and are based on molecules that bear no structural resemblance. What has been missing so far is a systematic analysis of Δ*G*^Φ^ between racemic crystal phases and their enantiopure counterparts for structurally related compounds. Such analysis might not only enable the systematic discovery of crystal structures that can be kinetically stabilized but may also guide rational experimental design to systematically exploit nonequilibrium conditions for destabilizing racemic compounds into their (kinetic) conglomerate counterparts (Figure 1c).

**Figure 2:**
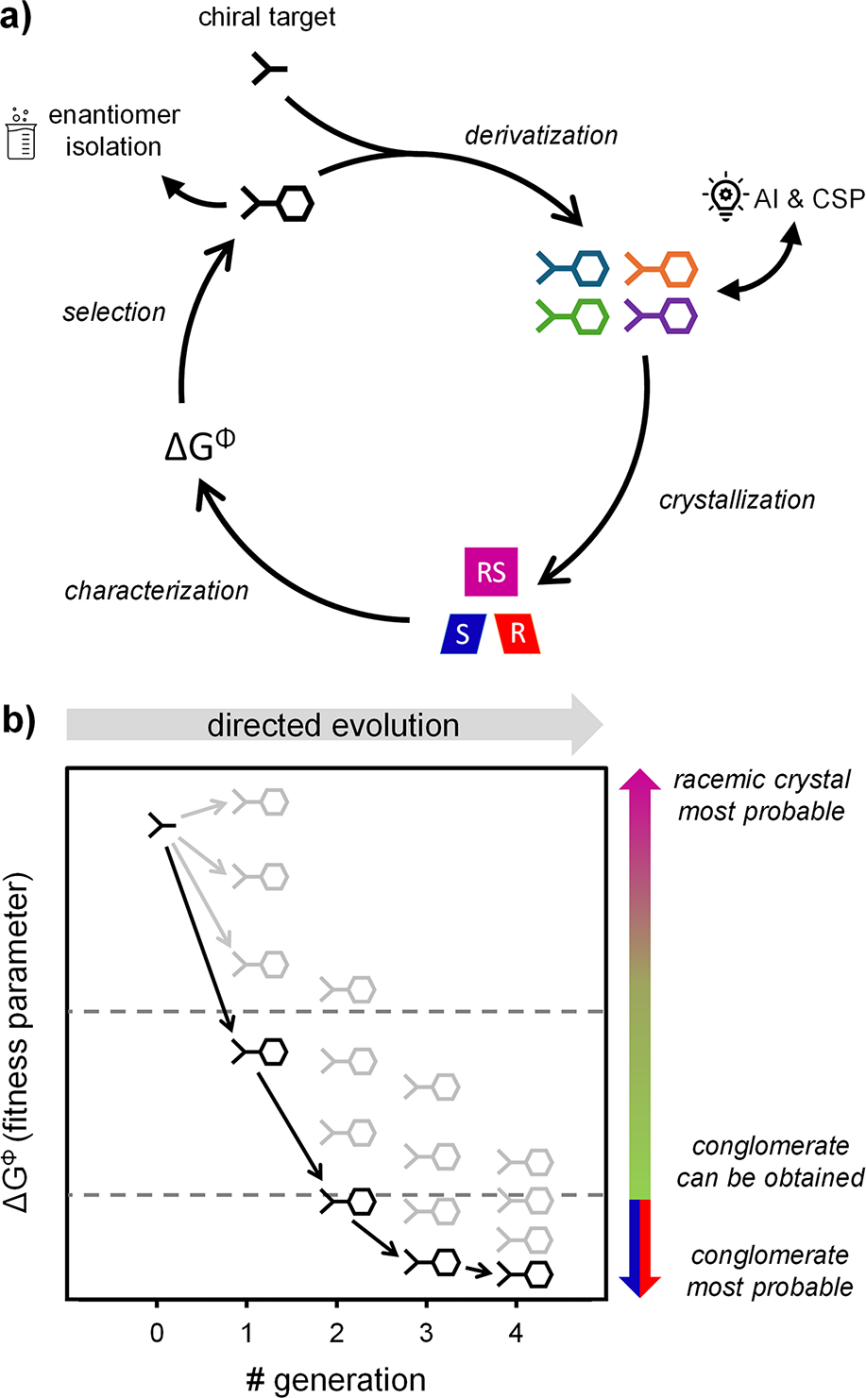
Concept of directed evolution for (kinetic) conglomerate discovery and rational library design. (a) Development of (kinetic) conglomerates of chiral targets by iterate cycles of chemical derivatization, crystallization, characterization, and selection. Δ*G*^Φ^ serves as a fitness parameter for selecting the input for the next generation. Artificial intelligence (AI) or crystal structure prediction (CSP) could synergistically inform the choice of derivatives, leveraging the results from previous generations. (b) Subsequent generations of target derivatives systematically evolve toward lower Δ*G*^Φ^. Due to diminishing returns with each additional iteration, the fitness parameter typically plateaus following a power law or exponential decay.^31,32^ Such directed evolution quickly discovers (meta)stable enantiopure crystal phases for isolating target enantiomers.

Motivated by these insights, we here systematically analyze Δ*G*^Φ^ between racemates and their enantiopure counterparts for two libraries of biorelevant target molecules with different chemical modifications. These libraries are found to exhibit a broad and continuous distribution for Δ*G*^Φ^, in which similar chemical modifications are grouped together. Akin to directed evolution in catalysis,^28^ we foresee that the relationship between Δ*G*^Φ^ and the chemical structure can be exploited by systematically selecting chemical modifications with the lowest Δ*G*^Φ^ to guide the synthesis of the next generation (Figure 2).^29−31^ Such an evolutionary method may efficiently identify metastable enantiopure crystal phases for isolating desired enantiomers under nonequilibrium conditions. Analysis of over a hundred chiral molecules in the literature supports that our findings are general and more than 50% of all chiral molecules are prone to be isolated in enantiopure form under nonequilibrium conditions (Figure 1c).

## Results & Discussion

**Figure 3:**
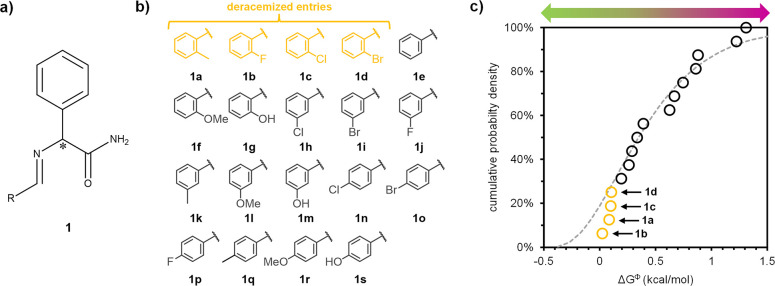
Analysis of free energy differences (Δ*G*^Φ^) between racemic (*RS*) and enantiopure (*R*) crystals of a library with common chiral center (full data in the SI). (a) Schiff-base derivatives of phenylglycinamide (**1**), chiral center indicated with *. (b) Synthesized library entries for **1**. (c) Cumulative probability distribution of free energy differences shows a wide variation in Δ*G*^Φ^ (dotted gray line is fitted gamma distribution based on the literature data set in Figure 5b). Low Δ*G*^Φ^ entries **1a**–**d** (yellow) were successfully deracemized.

We investigated Δ*G*^Φ^ between enantiopure and racemic crystal forms for a library of chemically analogous chiral molecules. As a chiral core, we select the Schiff base of phenylglycine amide **1**, an amino acid derivative that serves as a building block in several pharmaceutical compounds, and which has previously been deracemized as conglomerate **1a**.^33,34^ These Schiff base derivatives can be formed straightforwardly from aldehydes to yield a library (Figure 3a).^35^ Following this procedure, a library of 19 derivatives, enantiopure as well as the racemic crystal form, with the same chiral core was obtained (Figure 3b).

We determine the Δ*G*^Φ^ for each pair of enantiopure and racemic crystal forms in the library. Differential scanning calorimetry (DSC) provides the melting points and heats of fusion for both crystal forms from which we compute Δ*G*^Φ^ (see the SI for details). Figure 3c shows the cumulative probability density of Δ*G*^Φ^. Although small, library **1** already displays a broad distribution of Δ*G*^Φ^, ranging from close to 0 to 1.5 kcal/mol. The stable conglomerate **1a** and derivatives **1b,c**—both having been identified as racemic compounds but deracemized previously^17^—group together with similar values of Δ*G*^Φ^ ≈ 0.1 kcal/mol. This grouping is consistent with our expectation that thermodynamically stable racemic compound entries with low Δ*G*^Φ^ values may be suitable for conversion into kinetic conglomerates.

To explore the predictive potential of Δ*G*^Φ^ further, we attempt the deracemization of **1d**, since **1d** is the next entry in the ascending order of Δ*G*^Φ^ (Figure 3c). A slurry of racemic **1d** was prepared, racemization was initiated using a base, and the mixture was subsequently seeded with enantiopure (*R*)-**1d** (see the SI for details). After 3 h of attrition, complete conversion of (*RS*)-**1d** into enantiopure (*R*)-**1d** was observed. This successful deracemization confirms that this compound had formed a (metastable) conglomerate and that Δ*G*^Φ^ can be used to predict the conversion into enantiopure crystals.

Entries **1a**–**d** not only show similar Δ*G*^Φ^ values but also have similar crystal structures,^36^ with all crystal structures sharing a common hydrogen bonding motif. From a molecular structure, the possible crystal structures can be predicted, for which, in turn, one can predict a corresponding Δ*G*^Φ^. However, our data suggest that Δ*G*^Φ^ could even be directly predicted from the molecular structure (without intervening considerations in the crystal structures). Revealing such a relationship would enable the rational and methodical library design of molecules with low Δ*G*^Φ^.

Akin to directed evolution in catalysis,^28^ we envisage that iterative selection of low Δ*G*^Φ^ molecules can systematically direct the design of modifications around a chiral center toward low Δ*G*^Φ^. Because of its continuous character as opposed to the binary classification of conglomerates and racemic compounds, we foresee that Δ*G*^Φ^ can be a convenient fitness parameter in directed evolution.

**Figure 4:**
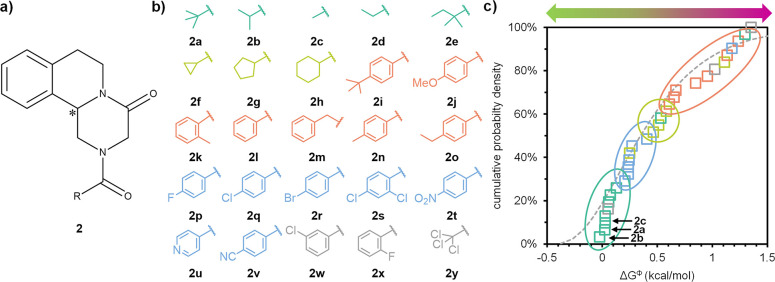
Potential of library design by directed evolution. (a) Praziquantel derivatives **2**, chiral center indicated with *. (b) Synthesized library entries for **2**, classified as alkyls (dark green), carbocycle (light green), aromatic alkyls (orange), halogen, and other substituted aromatics (light blue), nonclassified (gray). (c) Cumulative probability distribution of free energy differences (full data in the SI; dotted gray line is fitted gamma distribution based on the literature data set in Figure 5b). Derivatives cluster by chemical classification (colored ellipses), enabling rational library design by directed evolution.

To assess the potential of such an evolutionary approach to molecular design, a library based on the chiral core of praziquantel **2** was synthesized (Figure 4a). Praziquantel (**2h**) is a racemic drug against parasitic worms, and there is wide interest in isolating the bioactive (*R*)**-2h** enantiomer.^37−39^ To investigate trends in chemical structures and Δ*G*^Φ^ systematically, we prepared 25 derivatives that group into four distinct modification classes (Figure 4b): alkyls (**2a**–**e**); carbocycles (**2f**–**h**); aromatic alkyls (**2i**–**o**); aromatic halides and other substituted aromatics (**2p**–**v**); and three unclassified derivatives (**2w**–**y**).

We determined and plotted the cumulative probability density distribution, Δ*G*^Φ^ (Figure 4c). With few exceptions, library entries cluster along Δ*G*^Φ^ according to the predetermined modification classes, enabling an evolutionary strategy for library design (Figure 2). Specifically, starting with only four entries (one per modification class) as the first generation, the alkyl derivative can be immediately identified as the most promising, since that entry shows the lowest Δ*G*^Φ^. Subsequently preparing a second generation of four additional alkyl derivatives already yields stable conglomerate **2a**. Hence, rather than preparing 25 quasiarbitrary derivatives, we can find conglomerates and low Δ*G*^Φ^ entries within just two generations and with less than a third of the total number of library entries (8 instead of 25), showing the potential of library design through directed evolution.

The clustering of chemical classes not only enables library design through directed evolution but also may group racemic compounds within the Δ*G*^Φ^-distribution that are suitable for isolating enantiopure crystals through kinetically stabilized conglomerates. To investigate this idea, we explore whether racemic compounds **2b** and **2c**, which are situated in the same low Δ*G*^Φ^-region as the known stable conglomerate **2a**, can be isolated as enantiopure crystals. To this aim, we prepare supersaturated racemic solutions of **2b** and **2c**, seed with (*R*)-**2b** and (*R*)-**2c**, respectively, and obtain the desired enantiomers in good yield and enantiopurity (>95% ee, see the SI for details).

**Figure 5:**
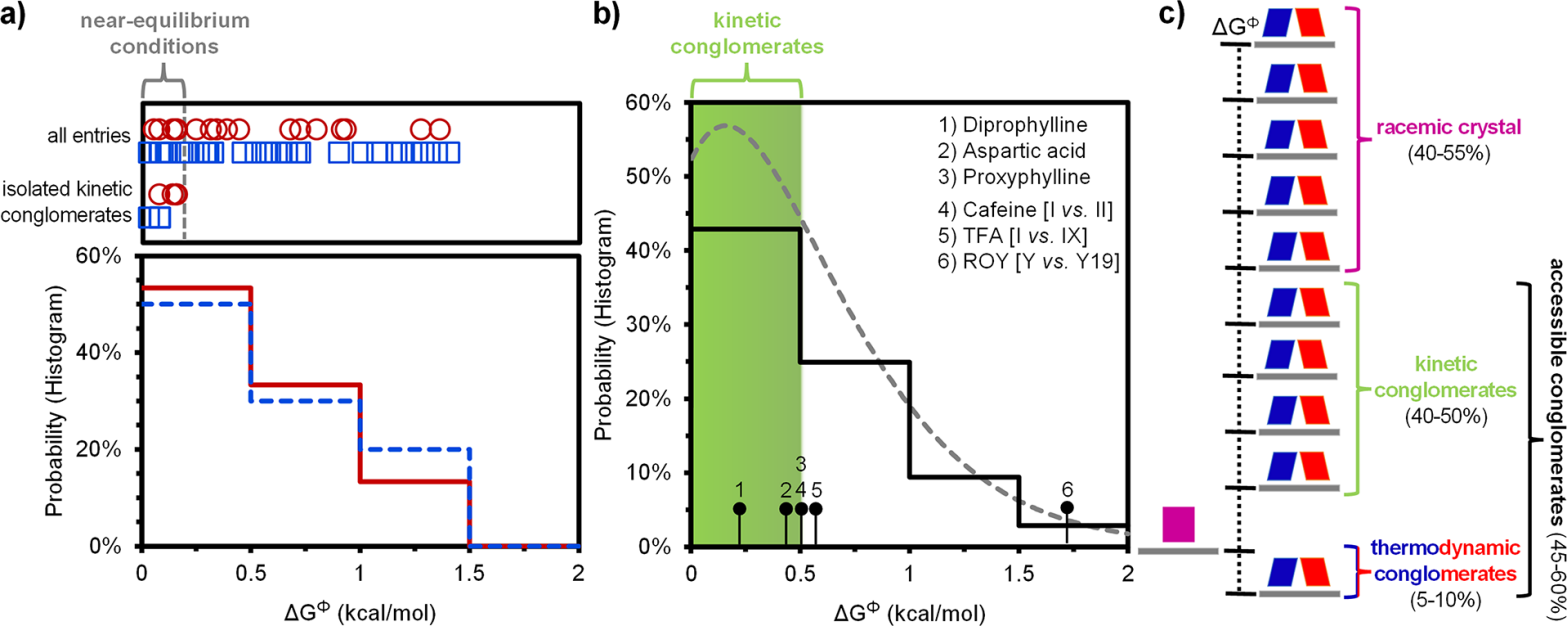
Generality of trends, and potential of nonequilibrium conditions for isolating enantiopure crystals. (a) Despite their chemical differences, library entries for **1** (red solid histogram) and **2** (blue dashed histogram) show similar probability distributions. Entries isolated as kinetic conglomerates under near-equilibrium conditions fall within Δ*G*^Φ^ < 0.2 kcal/mol region. (b) Probability histogram of Δ*G*^Φ^ for 100+ unrelated chiral molecules from the literature (black solid histogram). Histograms from libraries **1**, **2**, and literature data are similar and well-described by the same gamma-distribution (gray dotted line), supporting the generality of these trends. Region of Δ*G*^Φ^ < 0.5 kcal/mol (shaded green) contains kinetic conglomerates (1–3) and unstable polymorphs (4,5), which indicates that ca. 40–50% of the racemic compounds may be kinetically stabilized as enantiopure crystals under near- or far-from-equilibrium conditions. (c) Predicted distribution was between thermodynamic conglomerates, kinetic conglomerates, and racemic crystals.

The chemical core of both libraries is very different: **1** is small and flexible and can undergo H-bonding, whereas **2** is large and stiff without possibilities for H-bonding.^40^ To understand how these differences impact the distribution of Δ*G*^Φ^, we plot the probability histograms of Δ*G*^Φ^ for **1** and **2** (Figure 5a). Comparison of both histograms shows that despite the difference in molecular structure their distribution in Δ*G*^Φ^ is strikingly similar. Also, for both libraries, we find that near-equilibrium conditions already allow for the straightforward isolation of enantiopure crystals from racemic compounds (when Δ*G*^Φ^ < 0.2 kcal/mol, Figure 5a). These commonalities prompt two questions. First, how general are these trends? Second, how much further can we push the threshold of Δ*G*^Φ^ for which racemic compounds convert into kinetic conglomerates by exploiting far-from-equilibrium conditions?

To address the question of generality, we collect thermodynamic data for more than a hundred chiral organic racemic compounds that have been previously investigated (see the SI).^11,41^ This literature catalog of molecules is very diverse, ranging from salts to molecules with multiple chiral centers and covering a wide breadth of functional groups featuring several heteroatoms (S, N, and O). Moreover, in contrast to our two libraries, the entries in the literature set are—to a large extent—structurally not related, thus forming a representative reference set for assessing generality. We find that the literature data are well-described by a gamma distribution (Figure 5b). A statistical comparison (Kolmogorov–Smirnov test) shows that both libraries **1** and **2** follow the same gamma distribution (see SI), as visualized in Figures 3c and 4c. These similarities suggest that the trends for the two libraries can be generalized to a large diverse set of unrelated chiral organic molecules.

Based on this analysis, we also assess the general potential of nonequilibrium conditions to kinetically stabilize enantiopure crystals. We identify three chiral molecules (diprophylline, aspartic acid, proxyphylline) for which the racemic compound has previously been kinetically converted to enantiopure crystals,^15,18,19^ calculate their Δ*G*^Φ^ (0.24, 0.45, and 0.48 kcal/mol respectively), and mark them for comparison with the energy difference distribution in Figure 5b. We realize that all three conversions require crystallization conditions that favor kinetic phases, suggesting that far-from-equilibrium conditions are essential.

Δ*G*^Φ^ of these compounds is close to the thermal energy *k*_*B*_*T* (0.6 kcal/mol), suggesting that transitions between crystal phases with such energy differences (Δ*G*^Φ^ ≤ 0.5 kcal/mol) are kinetically probable. This idea is consistent with observations beyond chiral crystallization, where polymorphic transitions are often reported when energy differences are below 0.5 kcal/mol.^22,24^ Notable examples include caffeine (Δ*G*^Φ^ = 0.5 kcal/mol)^42^ and tolfenamic acid (TFA, Δ*G*^Φ^ = 0.55 kcal/mol)^43^ (Figure 5b). For some reported transformations the energy differences are even much larger, as exemplified by the archetypical polymorphic system known as ROY, with a Δ*G*^Φ^ as large as 1.7 kcal/mol).^23,44^ Hence, we estimate that 40–50% of thermodynamically stable racemic compounds (Δ*G*^Φ^ ≤ 0.5 kcal/mol) can likely be kinetically obtained as enantiopure crystals under near- or far-from-equilibrium conditions (Figure 5b). Additionally, 5–10% of chiral compounds already crystallize as stable conglomerates. Consequently, we predict that 45–60% of all chiral compounds can be isolated as desired enantiomers through crystallization under either equilibrium or nonequilibrium conditions (Figure 5c).

## Conclusions

In summary, by systematically investigating the energy differences between the racemic and enantiopure crystal forms of structurally related molecules, we outline how combining directed evolution and combinatorial chemistry enables the expedient discovery of metastable enantiopure crystal phases that can be kinetically stabilized for isolating enantiomers of the desired handedness. Until now, hindered by thermodynamic limitations, it was generally understood that merely 5–10% of chiral molecules could be accessed as enantiopure crystals. In contrast, we here estimate that at least 50 to 65% of all chiral molecules are accessible as enantiopure crystals through nonequilibrium crystallization.

These insights can be directly implemented for the rational discovery of chiral compounds that can be separated by crystallization. Even though the change of only a single atom can drastically change the stability of crystal phases, we observe the clustering of similar derivatives within a library, which enables methodological library design. Specifically, we envision the autonomous construction of chemical libraries in self-driven laboratories,^45,46^ following an iterative manner, in which a rapid assessment of Δ*G*^Φ^ serves as a diagnostic guide for the design of new library entries and the efficient discovery of targets for resolution or deracemization. Analogously to directed evolution in catalysis,^29−31^ we propose to synthesize a small library with very diverse entries that are ranked according to Δ*G*^Φ^ as a fitness parameter, after which the most favorable entry is selected for synthesis of the next generation of entries. This evolutionary strategy prevents only unfavorable zones with high Δ*G*^Φ^ being screened and instead iterates toward favorable low Δ*G*^Φ^ within only a few cycles. We foresee that making informed design choices may be further aided by integrating crystal structure prediction (CSP) methodologies.^47,48^

For entries with low Δ*G*^Φ^, we have shown here that enantiopure crystals can successfully be isolated from racemic mixtures by applying near-equilibrium conditions. The key next step is to systematically exploit far-from-equilibrium conditions, under which crystallization rates—instead of thermodynamic stabilities alone—determine which crystalline phase is favored such that for instance, the desired kinetic conglomerate grows faster than the undesired racemic compound. Alternatively, specific nonequilibrium conditions can be exploited to suppress the nucleation and growth rate of stable racemic compound crystals, such that the desired enantiopure crystals can be isolated. Importantly, the crystallization process offers a large parameter space that can be exploited to achieve these favorable rates of nucleation and growth, ranging from choice of solvent, confinements such as microdroplets, and (chiral) additives to temperature gradients and mechanochemistry. Indeed, mechanical grinding and temperature gradients have also been used to achieve deracemization of solid phases,^1−3,49^ suggesting possibilities to yield nonequilibrium conditions that destabilize racemic compounds and simultaneously convert racemic (or partially enriched) solid phases into the desired enantiomer. Ultimately, especially with the rise of machine learning techniques and self-driven laboratories to design and execute the synthesis of chiral molecules,^50^ evaluating the potential for resolving or deracemizing key intermediates should become an integrated aspect of synthesizing enantiopure molecules.

## Supporting Information Available

Supporting Information: experimental and methodological details (synthesis procedures, DSC characterization, computation of Δ*G*^Φ^, deracemization and resolution procedures), DSC characterization data, deracemization, and resolution data (**1d**, **2b**, **2c**), literature data set, fitting of gamma distribution, and statistical tests (PDF)
