# Supplementary material for: Enantiopurity by Directed Evolution of Crystal Stabilities and Nonequilibrium Crystallization
Source: J Am Chem Soc. 2025 Feb 25;147(10):8864–70. doi: 10.1021/jacs.5c00569 (PMC11912332; doi:10.1021/jacs.5c00569)
Supplement: Supplementary file 1 — ja5c00569_si_001.pdf [file ja5c00569_si_001.pdf]

Supplementary Information

Enantiopurity by directed evolution of crystal stabilities and non-equilibrium crystallization

Clément Pinetre[a†], Sjoerd W. van Dongen[b†], Clément Brandel[a*], Anne-Sophie Léonard[b], Maxime D. Charpentier[c], Valérie Dupray[a], Kasper Oosterling[d], Bernard Kaptein[e], Michel Leeman[d], Richard M. Kellogg[f], Joop H. ter Horst[c,g*], Willem L. Noorduin[b,h*]

a) Univ Rouen Normandie, Normandie Univ, SMS , UR 3233, F-76000 Rouen, France
b) AMOLF, Science Park 104, Amsterdam 1098 XG, The Netherlands
c) EPSRC Future Continuous Manufacturing and Advanced Crystallisation Research Hub, c/o Strathclyde Institute of Pharmacy and Biomedical Sciences, University of Strathclyde, Glasgow, G1 1RD, U.K.
d) Symeres, Kadijk 3, 9747 AT Groningen, The Netherlands
e) InnoSyn, Urmonderbaan 22, 6167 RD Geleen, The Netherlands
f) Kellogg Beheer B.V., Zernikepark 12, Unit 1.31, 9747 AN Groningen, The Netherlands
g) Tiofarma, Hermanus Boerhaavestraat 1, 3261 ME Oud-Beijerland, The Netherlands
h) Van 't Hoff Institute for Molecular Sciences, University of Amsterdam, Science Park 904, Amsterdam 1090 GD, The Netherlands

† these authors contributed equally
* corresponding authors: clement.brandel@univ-rouen.fr, JtHorst@tiofarma.nl, noorduin@amolf.nl

Table of Contents

1. General Materials & Methods

2. Computation of ΔG^{ϕ}

3. Library **1**

   3a. Synthesis of library **1**

   3b. DSC characterization of library **1**

   3c. Deracemization of **1d**

4. Library **2**

   4a. Synthesis of library **2**

   4b. DSC characterization of library **2**

   4c. Resolution of **2b** and **2c**

5. Literature dataset

   5a. Thermochemical data compiled from literature

   5b. Fitting of the gamma distribution

   5c. Statistical tests

1. General Materials & Methods

Chemicals

Library **1** was kindly provided by InnoSyn (Geleen, NL) in both racemic and enantiopure form and used as is. For the deracemization of **1d**, the racemization catalyst was 1,8-Diazabicyclo[5.4.0]undec-7-ene (DBU, Across Organics) and the solvent was methanol sourced from Thermo Scientific Chemicals (99.9%, for spectroscopy). Library **2** was kindly provided by Symeres (Groningen, NL) in both racemic and enantiopure form and used as is. For the preferential crystallization of **2b** and **2c**, 2-propanol from VWR chemical (≥ 99%, HPLC grade) was used.

DSC characterization method

Thermal analyses were performed on a Netzsch DSC 214 Polyma or a DSC Q20 V24.11 Build 124 apparatuses. DSC runs were performed with ~4−5 mg of the solid sample in pierced aluminium pans and using heating rates of 5 or 10 $K.min^{-1}$. The atmosphere of the analyses was regulated by a nitrogen flux (40$mL.min^{-1}$). The Netzsch Proteus Software was used for data processing.

HPLC analysis method

For the determination of enantiomeric composition of samples from library **1**, chiral HPLC analyses were performed using an Agilent Technologies Infinity 1260 HPLC system. HPLC analysis was performed on a chiral column (CHIRALPAK IA (250 x 4.6 mm, 5μm)) with a mobile phase consisting of n-heptane and 2-propanol, where the eluent is mixed in a 7:3 ratio (heptane:IPA). All solvents used in these HPLC analysis (n-heptane, 1-propanol) were HPLC grade (≥ 99%) and obtained from VWR chemicals. The flow rate was 0.7 mL/min, injection volume 4 μL, and detection was performed by UV-detector (wavelength: 220 nm). Each run had a total time of 12 minutes.

For the determination of enantiomeric composition of samples from library **2**, chiral HPLC analyses were performed using an Ultimate 3000 HPLC (ThermoFisher Scientific). HPLC analysis was performed on a Phenomenex Lux Cellulose 4 column (3.0 x 150 mm x 3 μm), Chiracel OJ-H, OD-H columns (4.6 x 250 mm, 5μm) or CHIRALPAK IC column (4.6 x 250 mm, 5μm) with a UV detection at a wavelength of 220 nm with a mobile phase consisting of n-heptane and 2-propanol at flow rate of 1 mL.min−1. All solvents used in these HPLC analysis (n-heptane, 1-propanol) were HPLC grade (≥ 99%) and obtained from VWR chemicals.

2. Computation of ΔG^{ϕ}

Melting point temperature and enthalpy of fusion were measured for the racemic (T_m^{RS} and ΔH_f^{RS}) and enantiopure crystals (T_m^{R} and ΔH_f^{R}) using DSC. Using these data, the free energy difference (ΔG^{ϕ}) was computed following previously developed approaches by Collet and Wilen:[1]

$$\Delta G^{\phi} = R(T_m^R - T_m^{RS})\ln 2 + \Delta H_f^R \left(1 - \frac{T_m^{RS}}{T_m^R}\right) - \Delta H_f^{RS}\left(\frac{T_m^R}{T_m^{RS}} - 1\right), \quad \text{(eq. 1)}$$

where *R* is defined as the gas constant.

3. Library 1

3a. Synthesis of library 1

The library **1** composed of Schiff-base derivatives of phenylglycinamide was provided by Innosyn and synthesized following the literature procedure.[2] In short, phenylglycinamide was first obtained from phenyl glycine. Subsequently, phenylglycinamide was reacted with a series of aldehydes to give library **1** (Table S1).

3b DSC characterization of library 1

Racemic and enantiopure solids from library **1** were analysed using DSC following the procedure described in section 1. From the DSC measurements we obtained the melting point and enthalpy of fusion of the racemic solid (T_m^{RS} and ΔH_f^{RS}) and the enantiopure solid (T_m^{R} and ΔH_f^{R}). Using equation 1, we computed the free energy difference (ΔG^{ϕ}) (Table S1).

Table S1: DSC measurements of melting temperatures and enthalpies of fusion for racemates (T_m^{RS} and ΔH_f^{RS}) and enantiopure solids (T_m^{R} and ΔH_f^{R}) of library **1**, and the computed free energy differences (ΔG^{ϕ}).

Entry	Compound	T_m^{RS} (K)	ΔH_f^{RS} (kcal/mol)	T_m^{R} (K)	ΔH_f^{R} (kcal/mol)	ΔG^{ϕ} (kcal/mol)
**1b**	(E)-2-((2-fluorobenzylidene)-amino)-2-phenylacetamide	403	-	430	8.39	0.06
**1a**	(E)-2-((2-methylbenzylidene)-amino)-2-phenylacetamide	430	8.20	453	9.59	0.13
**1c**	(E)-2-((2-chlorobenzylidene)-amino)-2-phenylacetamide	427	9.32	451	9.00	0.19
**1d**	(E)-2-((2-bromobenzylidene)-amino)-2-phenylacetamide	413	-	436	8.57	0.25
**1e**	(E)-2-(benzylideneamino)-2-phenylacetamide	399	7.63	419	7.40	0.31
**1o**	(E)-2-((4-bromobenzylidene)-amino)-2-phenylacetamide	430	9.70	446	9.25	0.38
**1n**	(E)-2-((4-chlorobenzylidene)-amino)-2-phenylacetamide	413	8.41	427	8.41	0.44
**1i**	(E)-2-((3-bromobenzylidene)-amino)-2-phenylacetamide	396	8.41	407	8.34	0.50
**1h**	(E)-2-((3-chlorobenzylidene)-amino)-2-phenylacetamide	390	8.80	397	7.89	0.56
**1f**	(E)-2-((2-methoxybenzylide- ne)-amino)-2 phenylacetamide	461	7.31	462	10.07	0.63
**1j**	(E)-2-((3-fluorobenzylidene)-amino)-2-phenylacetamide	393	7.26	386	7.26	0.69
**1s**	(E)-2-((4-hydroxybenzylidene)-amino)-2-phenylacetamide	425	4.86	410	4.38	0.75
**1l**	(E)-2-((3-methoxybenzylidene)-amino)-2-phenylacetamide	415	9.49	402	12.12	0.81

**1g**	(E)-2-((2-hydroxybenzylidene)-amino)-2-phenylacetamide	429	7.90	413	7.78	0.88
**1r**	(E)-2-((4-methoxybenzylidene)-amino)-2-phenylacetamide	404	6.86	364	5.58	0.94
**1p**	(E)-2-((4-fluorobenzylidene)-amino)-2-phenylacetamide	427	9.40	394	9.40	1.00
**1q**	(E)-2-((4-methylbenzylidene)-amino)-2-phenylacetamide	403	7.96	428	9.47	-

Remarks on Table S1

1b) Exothermic phase transition upon melting. ΔG^{Φ} has been estimated following its linear correspondence to $\Delta T^{(RS-R)}$, as shown in Figure S1.

1d) Exothermic phase transition upon melting. ΔG^{Φ} has been estimated following its linear correspondence to $\Delta T^{(RS-R)}$, as shown in Figure S1.

1k) Could not be crystallized, was not characterized by DSC, and is therefore not included in Table S1.

1m) Could not be crystallized, was not characterized by DSC, and is therefore not included in Table S1.

1q) Stable racemic compound which shows non-ideal thermodynamic behavior due to interactions between the two enantiomers in the melt and solution phase. This non-ideality invalidates the assumptions made underlying eq. 1 so that ΔG^{Φ} would not reflect the actual energy difference between the crystal phases.

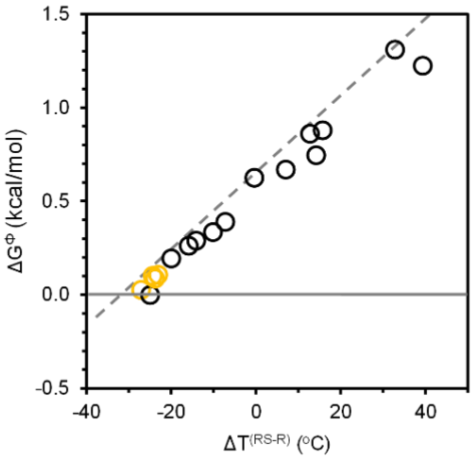

Figure S1: Difference in free energy between the racemate and the enantiopure form (ΔG^{Φ}) as a function of the difference in the temperature between the racemate and the enantiopure form $\Delta T^{(RS-R)}$ (°C) showing a linear relationship. Fitted equation: $\Delta G^{\Phi} \approx 0.02 \cdot \Delta T^{(RS-R)} + 0.6$.

3c. Deracemization of 1d

A 7 mL vial (27150-U Supelco, Merck) was charged with 20 mg of (*R*)-**1d** and 100 mg of racemate (Figure S2a), to yield a starting composition of the solid with 17% enantiomeric excess in *R*. Subsequently, a stirring bar (6x3mm cylindrical PTFE stirring bar, VWR), 1 g of glass beads (borosilicate, 2 mm diam.), and 1 mL of methanol (MeOH) were added to the solid, and the vial was briefly vortexed. To start the conversion, 20 μL of DBU was added and the slurry was stirred vigorously for 3 hours. After these 3 hours, the enantiomeric excess of the solid was determined by casting the slurry on top of filter paper laid down on a P5 glass filter connected to a vacuum filtration set-up (whilst under active vacuum). A sample of the solid (~1 mg) was

then taken using a Pasteur pipette, dissolved in 1.5 mL of 2-propanol by ultrasonication, and submitted to HPLC analysis (Figure S2b). The HPLC analysis showed a final composition of the solid of 97.9% enantiomeric excess in *R*, implying virtually full deracemization of **1d** towards the desired *R*-enantiomer.

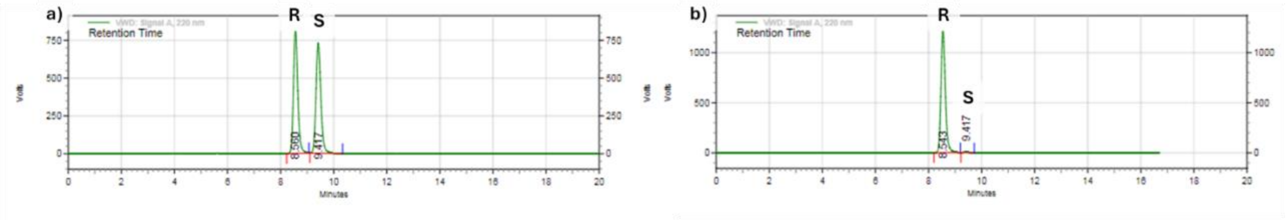

Figure S2: HPLC chromatograms of **(a)** the racemate of **1d**, showing peaks for both (*R*)-**1d** and (*S*)-**1d** in equal amounts, and **(b)** the solid obtained after the deracemization procedure, showing virtually only (*R*)-**1d** remaining.

4. Library 2

4a Synthesis of Library 2

The library **2** composed Praziquantel derivatives was provided by Symeres and synthesized following the literature procedure.[3] In short, different library entries were created by reacting praziquanamine with various acyl chlorides to give library **2** (Table S2).

4b. DSC characterization of library 2

Racemic and enantiopure solids from library **2** were analysed using DSC following the procedure described in section 1. From the DSC measurements we obtain the melting point and enthalpy of fusion of the racemic solid (T_m^{RS} and ΔH_f^{RS}) and the enantiopure solid (T_m^R and ΔH_f^R). Using equation 1, we compute the free energy difference (ΔG^{ϕ}) (Table S2).

Table S2. DSC measurements of melting temperatures and enthalpies of fusion for racemates (T_m^{RS} and ΔH_f^{RS}) and enantiopure solids (T_m^R and ΔH_f^R) of library **2**, and the computed free energy differences (ΔG^{ϕ}).

Entry	Compound	T_m^{RS} (K)	ΔH_f^{RS} (kcal/mol)	T_m^R (K)	ΔH_f^R (kcal/mol)	ΔG^{ϕ} (kcal/mol)
**2a**	2-pivaloyl-1,2,3,6,7,11b-hexahydro-4H-pyrazino[2,1-a]isoquinolin-4-one	425	4.0	454	4.4	0.03
		428	3.7			0.08
**2b**	2-isobutyryl-1,2,3,6,7,11b-hexahydro-4H-pyrazino[2,1-a]isoquinolin-4-one	388	2.8	419	3.7	-0.02
		391	3.1			0.03
**2c**	2-acetyl-1,2,3,6,7,11b-hexahydro-4H-pyrazino[2,1-a]isoquinolin-4-one	415	3.1	454	3.2	0.03
		417	3.3			0.06
		421	2.7			0.13
**2d**	2-propionyl-1,2,3,6,7,11b-hexahydro-4H-pyrazino[2,1-a]isoquinolin-4-one	421	4.3	423	3.9	0.53

**2e**	2-(2,2-dimethylbutanoyl)-1,2,3,6,7,11b-hexahydro-4H-pyrazino[2,1-a]isoquinolin-4-one	438	4.3	400	3.3	1.30
**2f**	2-(cyclopropanecarbonyl)-1,2,3,6,7,11b-hexahydro-4H-pyrazino[2,1-a]isoquinolin-4-one	400	2.5	422	2.8	0.25
		418	4.9			0.51
		422	3.0			0.58
**2g**	2-(cyclopentanecarbonyl)-1,2,3,6,7,11b-hexahydro-4H-pyrazino[2,1-a]isoquinolin-4-one	407	4.0	413	3.1	0.47
**2h**	2-(cyclohexanecarbonyl)-1,2,3,6,7,11b-hexahydro-4H-pyrazino[2,1-a]isoquinolin-4-one	414	4.1	384	2.8	1.11
**2i**	2-(4-(tert-butyl)benzoyl)-1,2,3,6,7,11b-hexahydro-4H-pyrazino[2,1-a]isoquinolin-4-one	476	4.3	458	4.1	0.96
**2j**	2-(4-methoxybenzoyl)-1,2,3,6,7,11b-hexahydro-4H-pyrazino[2,1-a]isoquinolin-4-one	491	4.9	482	6.3	0.85
**2k**	2-(2-methylbenzoyl)-1,2,3,6,7,11b-hexahydro-4H-pyrazino[2,1-a]isoquinolin-4-one	394	3.0	389	2.5	0.61
**2l**	2-benzoyl-1,2,3,6,7,11b-hexahydro-4H-pyrazino[2,1-a]isoquinolin-4-one	438	4.2	408	2.5	1.14
**2m**	2-(2-phenylacetyl)-1,2,3,6,7,11b-hexahydro-4H-pyrazino[2,1-a]isoquinolin-4-one	394	3.0	389	2.5	0.61
**2n**	2-(4-methylbenzoyl)-1,2,3,6,7,11b-hexahydro-4H-pyrazino[2,1-a]isoquinolin-4-one	460	4.4	459	4.5	0.65
**2o**	2-(4-ethylbenzoyl)-1,2,3,6,7,11b-hexahydro-4H-pyrazino[2,1-a]isoquinolin-4-one	447	4.6	414	3.1	1.24
**2p**	2-(4-fluorobenzoyl)-1,2,3,6,7,11b-hexahydro-4H-pyrazino[2,1-a]isoquinolin-4-onecc	460	3.7	479	4.9	0.24
**2q**	2-(4-chlorobenzoyl)-1,2,3,6,7,11b-hexahydro-4H-pyrazino[2,1-a]isoquinolin-4-onebbr	491	5.9	510	6.3	0.21
**2r**	2-(4-bromobenzoyl)-1,2,3,6,7,11b-hexahydro-4H-pyrazino[2,1-a]isoquinolin-4-one	487	5.4	511	4.3	0.27
**2s**	2-(2,4-dichlorobenzoyl)-1,2,3,6,7,11b-hexahydro-4H-pyrazino[2,1-a]isoquinolin-4-one	411	3.0	431	3.7	0.24
**2t**	2-(4-nitrobenzoyl)-1,2,3,6,7,11b-hexahydro-4H-pyrazino[2,1-a]isoquinolin-4-one	485	4.8	498	5.0	0.41

**2u**	2-isonicotinoyl-1,2,3,6,7,11b-hexahydro-4H-pyrazino[2,1-a]isoquinolin-4-one	413	3.5	437	3.1	0.24
**2v**	4-(4-oxo-1,3,4,6,7,11b-hexahydro-2H-pyrazino[2,1-a]isoquinoline-2-carbonyl)benzonitrile	490	4.2	458	3.3	1.18
**2w**	2-(3-chlorobenzoyl)-1,2,3,6,7,11b-hexahydro-4H-pyrazino[2,1-a]isoquinolin-4-one	457	4.5	435	3.8	1.02
**2x**	2-(2-fluorobenzoyl)-1,2,3,6,7,11b-hexahydro-4H-pyrazino[2,1-a]isoquinolin-4-one	401	3.3	436	3.1	0.06
**2y**	2-(2,2,2-trichloroacetyl)-1,2,3,6,7,11b-hexahydro-4H-pyrazino[2,1-a]isoquinolin-4-one	459	4.2	416	2.8	1.35

Remarks on Table S2

2a) A racemic compound with a higher melting point than the reported conglomerate was detected. Further experiments highlighted the thermodynamic stability of the conglomerate at room temperature while the racemic solid phase becomes thermodynamically stable at elevated temperatures. ΔG^{Φ} has been calculated for the two solid phases.

2b) The racemic conglomerate and the racemic compound could be grown from its racemic mixture. Further experiments highlighted the thermodynamic stability of the racemic compound, at least for temperatures greater than -30 °C. ΔG^{Φ} has been calculated for the two solid phases.

2c) Two polymorphic forms of the racemic compound and the racemic conglomerate could be crystallized and characterized by DSC. ΔG^{Φ} has been calculated for the three solid phases.

2f) Three polymorphic forms of the racemic compound exhibiting an enantiotropic relationship.[4] ΔG^{Φ} has been calculated for the three solid phases.

4c. Resolution of 2b and 2c

SIPC method

~ 800 mg of racemic **2b** and **2c** was dissolved in 10 mL and 30 mL IPA using a 20 mL and 50 mL vials respectively by heating the resulting stirred mixture (cross-shaped PTFE magnetic stirring bars (BOHLC369-25, VWR) and standard stirring plate at 700rpm) until a clear solution is obtained (double-jacketed flasks connected to a circulating cryostat (F34-HE, Julabo)). The clear solution was then filter through 0.22 µm syringe filter and cooled to reach a supersaturation β = 2 (15 °C and 20 °C respectively) with respect to the solubility of the racemic compound (Figure S3) and seeded with 40 mg of pure enantiomer.

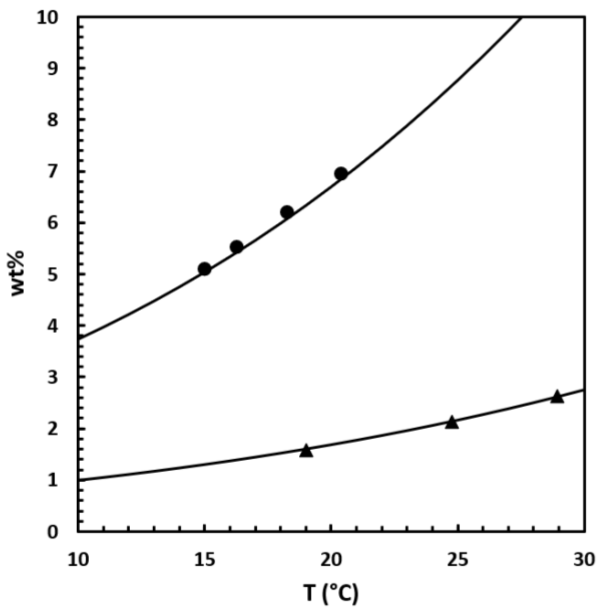

Figure S3: Temperature dependent solubility of **2b** (circle) and **2c** (triangle) in IPA. Solubility lines are theoretical modelling using Van't Hoff equation.

<u>Sampling methods and sample preparation</u>

For compound **2b** and **2c**, the chiral resolution via preferential crystallization was monitored by sampling ~ 0.5mL of the slurry from the crystallization flask using a single channel mechanical pipettor (613-0155, VWR). The solid and liquid phases were isolated from the collected suspension following two procedures :

- By casting it on top of filter papier laid down on glass filter connected to a vacuum filtration setup
- By centrifuge filtration using standard micro centrifuge (Fisherbrand) and 0.22µm cellulose acetate centrifuge tube filter (525-0017, VWR)

~1.5 mg of the solid phase was dissolved in 1.5 mL of a n-Heptane/IPA mixture (80/20; v/v) with subsequent ultrasonication while the liquid phase was diluted 9 times with the same solvent mixture prior to HPLC analyses.

<u>HPLC methods and SIPC monitoring</u>

Enantioselective HPLC analyses for compound 2b and 2c were performed on a Chiracel OD-H column (4.6 x 250 mm, 5µm) with a mobile phase consisting of n-heptane and 2-propanol (85/15 and 60/40 v:v respectively) at flow rate of 1 mL.min−1 with a UV detection at 220 nm. Each run had a total time of 60 minutes (Figure S4).

The crystallized mass was obtained by (i) weighing the total amount of enantiopure solid recovered or (ii) determining the concentration of the solute based on the calibration curve in Figure S5. The evolution of the crystallized mass and its associated enantiomeric excess (e.e.) are summarized in Table S3.

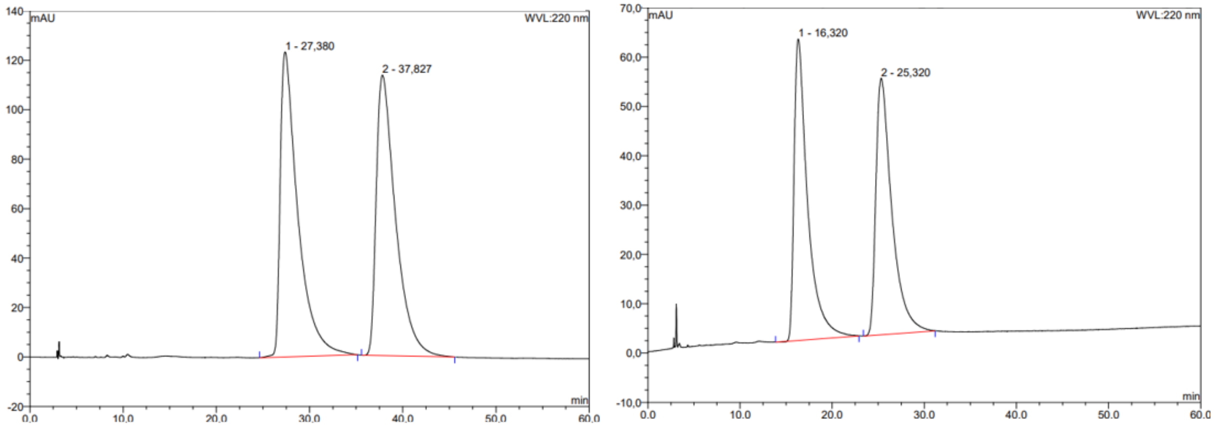

Figure S4: Typical chromatogram of racemic **2b** (left) and racemic **2c** (right). Retention time (min) for **2b**: (*R*)-**2b**: 27.38 (*S*)-**2b**: 37.83 and 2c: (*R*)-**2c**: 16.32 (*S*)-**2c**: 25.32.

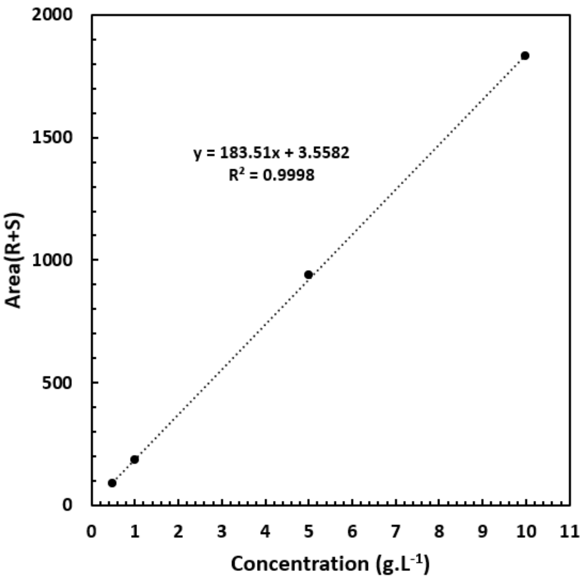

Figure S5: Calibration curve for derivative **2b**.

Table S3: SIPC monitoring result of compounds **2b** and **2c** giving the crystallized mass (mass of initial seed already deducted) and the enantiomeric excess of both the solid and liquid phases. The *e.e.* positive sign is arbitrary attributed to the (*R*) enantiomer.

	**2b**			**2c**		
Sampling time (min)	Crystallized mass (mg)	*e.e.* (%)		Crystallized mass (mg)	*e.e.* (%)	
		Solid	liquid		Solid	liquid
5	43.6	95.7	- 6.3	14.8	99.4	- 0.9
15	131.1	98.7	- 6.6	46.9	98.4	- 1.2
30	151.4	95.6	-7.6	77.0	98.9	- 1.5
60				87.1	97.2	- 1.9
120				96.8	94	- 2.4

5. Literature dataset

5a. Thermochemical data compiled from literature

The melting point and enthalpy of fusion of racemic solids (T_m^{RS} and ΔH_f^{RS}) and enantiopure solids (T_m^{R} and ΔH_f^{R}) were obtained from literature.[1,5,6] Using equation 1, we computed the free energy difference (ΔG^{ϕ}) (Table S3).

Table S3: Literature dataset of melting temperatures and enthalpy of fusion for racemates (T_m^{RS} and ΔH_f^{RS}) and enantiopure solids (T_m^{R} and ΔH_f^{R}), and computed difference in free energy (ΔG^{ϕ}).

No.	Compound	T_m^{RS} (K)	ΔH_m^{RS} (kcal/mol)	T_m^{R} (K)	ΔH_m^{R} (kcal/mol)	ΔG^{Φ} (kcal/mol)
**1**	(S,S)-3,5- Dimercaptoheptanedioic acid	355	9.40	429	5.00	-0.37
**2**	3,5-Dimercaptoheptanedioic acid	355	9.40	429	5.00	-0.37
**3**	11,12-Di(hydroxymethyl)-9,10-dihydro-9,10-ethanoanthracene	406	9.85	475	5.70	-0.27
**4**	2-(1-Naphthyl) propanoic acid	342	7.30	423	3.40	-0.18
**5**	9,10-Dimethyl-9,10 dihydro-9,10- ethano-11,12-dicarbomethoxyanthracene trans	393	9.80	465	4.50	-0.16
**6**	1-(Propan-2-ylamino)-3-(2-prop-2-enyphenoxy) propan-2-ol	299	8.51	331	5.68	-0.15
**7**	1-(Propan-2-ylamino)-3-(2-prop-2-enyphenoxy)propan-2-ol; hydrochloride	299	8.51	331	5.68	-0.15
**8**	4-Nitro-2-phenoxypropionic acid	362	7.46	412	5.00	-0.11
**9**	4-Nitro-2-phenoxy propionic acid	362	7.70	412	5.00	-0.10
**10**	(2S,3S)-2-Acetamido-N,3-dimethyl-pentanimde	482	6.48	525	9.11	-0.08
**11**	3-(4-Bromophenyl)-3-hydroxypropanoic acid	371	6.90	398	8.50	-0.07
**12**	4-[2-(3,5-Dioxopiperazin-yl)propyl] piperazine-2,6-dione	468	10.75	507	9.04	-0.07
**13**	(1R,2S)-2-Amino-1-phenyl-1-propanol	324	6.24	374	3.79	-0.06
**14**	(R,S)-N-Methylephedrine	336	6.36	361	7.30	-0.05
**15**	1-Butyl-N-(2,6-dimethylphenyl) piperidine-2-carboxamide	376	4.62	413	6.27	-0.04
**16**	1-Naphthalen-1-yloxy-3-(propan-2-ylamino)- propan-2-ol	344	9.75	367	8.37	-0.04
**17**	(S)-Fluoro-methyl-naphtalen-phenyl-1-ysilane	312	5.40	341	5.60	-0.04
**18**	2-(4-Methoxyphenyl)-1-phenylpropan-1-one	326	5.20	353	6.30	-0.03
**19**	3-(4-Chlorophenyl)-3-hydroxy- propanoic acid	357	6.70	385	7.10	-0.02
**20**	(1S,2S)-1,2-Dichloro-1,2-dihydro-acenaphthylene	339	4.90	375	5.10	-0.02
**21**	(1R,2S)-2-(Methylamino)-1-phenylpropan-1-ol	313	6.95	351	4.14	-0.02
**22**	1,5-Dichloro-9,10-dihydro-9,10-ethano anthracene	354	6.60	424	3.00	-0.01

**23**	4-Hydroxy-2-pyrrolidone	395	6.39	430	6.81	-0.01
**24**	2-Hydroxy-3-phenylpropanoic acid	368	7.29	395	7.29	0.00
**25**	3-Hydroxy-3-phenylpropanoic acid	366	7.10	391	7.80	0.01
**26**	1,7,7-Trimethyl-3-[(4-methoxyphenyl) methylidene]bicyclo[2.2.1]heptan-2-one	372	6.30	400	7.20	0.01
**27**	1,7,7-Trimethyl-3-[(4-methoxyphenyl) methylidene]bicyclo[2.2.1]heptan-2-one	372	6.31	400	7.20	0.01
**28**	(3-Fluorophenyl)-3-hydroxy propanoic acid	290	4.90	311	5.80	0.01
**29**	(1S,2S)-2-Methylamino-1-phenylpropan-1-ol;hydrosulfide	381	7.77	404	8.75	0.03
**30**	3-Hydroxy-2,2-dimethyl-3-phenylpropanoic acid	407	8.90	431	9.50	0.03
**31**	1-[2-(3,4-Dimethoxyphenyl) ethylamino]-3-(3-methylphenoxy)- propan-2-ol; hydrochloride	408	9.68	428	11.03	0.04
**32**	(1R,2R)-1,2-Diphenylethane-1,2-diol	394	7.20	420	8.00	0.06
**33**	Chloro-3-methyl-2-phenoxy propionic acid	360	7.30	392	5.30	0.06
**34**	2-Hydroxybutanedioic acid	373	7.63	402	6.33	0.06
**35**	(2-Chlorophenyl)(hydroxy)acetic acid	363	5.70	392	5.98	0.07
**36**	2-Phenoxypropionic acid	359	7.90	388	5.40	0.09
**37**	1,5-Dichloro-9,10-dihyro-9,10 ethano-11,12-dicarbomethoxyanthracene (exo)	424	8.80	465	5.55	0.09
**38**	2,2,3-Triphenylpentanoic acid	442	8.90	480	6.40	0.09
**39**	2-(4-(2-Methylpropyl)-phenyl) propanoic acid	320	6.06	346	4.47	0.10
**40**	1-[2-(3,4-Dimethoxyphenyl) ethylamino]-3-(3-methylphenoxy) propan-2-ol	348	10.97	361	10.57	0.12
**41**	2-(3-Chlorophenoxy)-propionic acid	368	7.90	386	7.10	0.17
**42**	2-(3-Chlorophenoxy) propanoic acid	368	7.90	386	7.10	0.17
**43**	(2-Fluorophenyl)-2-hydroxyacetic acid	362	7.30	389	4.78	0.17
**44**	(2S,3S)-Phenylglyceric acid	372	7.50	395	5.60	0.18
**45**	2-(2-Chlorophenoxy)-propionic acid	369	7.70	388	6.40	0.19
**46**	Dimethyl O,O-dibenzoyltartrate	409	11.70	423	11.00	0.21
**47**	2-[(2-Aminoethoxy)methyl]-4-(2-chlorophenyl)-ethoxycarbonyl-5-methoxycarbonyl-6-methyl-1,4-dihydropyridine	384	4.83	414	4.03	0.24
**48**	2-(Cyclohexanecarbonyl)-3,6,7,11b-tetrahydro-1H-pyrazino[2,1]isoquinolin-4-one	384	6.15	409	4.42	0.25
**49**	2-(3-Benzoylphenyl)propanoic acid	345	5.01	367	3.51	0.27
**50**	2-Amino-1-phenylpropan-1-ol; hydrosulfide	375	7.12	391	5.56	0.29
**51**	9,10-Dihydro-9,10ethano-11,12-dicarbomethoxyanthracene	363	5.60	380	4.00	0.32
**52**	2-(3-Chlorophenyl)-2-hydroxy acetic acid	367	5.01	380	5.07	0.33
**53**	2-(3,4-Dimethoxyphenyl)-5-[2-(3,4-dimethoxyphenyl)ethyl-methylamino]-2-propan-2-ylpentanenitrile; hydrochloride	408	15.39	417	9.72	0.37

**54**	(1S,2S)-2-Amino-1-phenyl-1-propanol; hydrochloride	446	6.92	469	4.84	0.38
**55**	(3-Chlorophenyl)(hydroxy)acetic acid	380	6.25	389	5.92	0.39
**56**	(1S,4S)-4-(3,4-Dichlorophenyl)-N-methyl-1,2,3,4-tetrahydronaphtalen-1-amine	340	5.12	343	5.86	0.42
**57**	3-Hydroxy-3-phenylpentanoic acid	379	8.40	384	7.40	0.43
**58**	2-(4-Bromophenoxy) propanoic acid	380	7.60	385	6.60	0.44
**59**	1,5-Dichloro-9,10-dihyro-9,10-ethano-11,12-dicarbomethoxyanthracene (endo)	427	6.30	436	5.90	0.47
**60**	2-(4-Chlorophenyl)-2-hydroxy acetic acid	379	5.48	384	4.69	0.47
**61**	2-(2-Oxopyrrolidin-1-yl)butanamide	389	7.44	393	6.51	0.48
**62**	3-(3-Bromophenyl)-3-hydroxypropanoic acid	350	1.62	349	5.70	0.49
**63**	3-(3-Bromo-3-phenyl)-3-hydroxy propanoic acid	350	6.40	349	5.70	0.50
**64**	1,5-Dichloro-11,12-dihydroxymethyl-9,10-dihydro-9,10-ethanoanthracene trans (endo)	435	7.90	441	4.70	0.54
**65**	2-Acetamido-N,4-dimethyl pentanamide	428	6.49	432	5.54	0.54
**66**	2-(4-Chlorophenyl)-2-hydroxy acetic acid	395	6.51	395	5.03	0.54
**67**	2-Methylamino-1-phenylpropan-1-ol	392	8.15	391	7.64	0.57
**68**	(2-Fluorophenyl)-3- hydroxy propanoic acid	348	6.50	342	5.40	0.58
**69**	5-O-Ethyl 3-O-methyl (4R)-4-(2,3-dichlorophenyl)-2,6-dimethyl-1,4-dihydropyridine-3,5-dicarboxylate	417	7.53	417	6.07	0.58
**70**	5-(2-Hydroxypropan-2-yl)-2-methylcyclohex-2-en-1-ol	383	6.18	379	5.54	0.59
**71**	N-(2,6-Dimethylphenyl)-1-ethylpiperidine-2-carboxamide	408	4.33	405	4.76	0.59
**72**	2-(6-Methoxynaphthalen-2-yl) propanoic acid	429	7.93	429	7.58	0.60
**73**	2-[4-(3-Oxo-1H-isoindol-2-yl) phenyl]-propanoic acid	483	10.02	486	12.35	0.61
**74**	N-(2,6-dimethylphenyl)-1-methylpiperidine-2-carboxamide	426	4.05	423	4.25	0.61
**75**	2-(Methylamino)-1-phenylpropan-1-ol	444	9.20	444	7.88	0.61
**76**	7-(2,3-Dihydroxypropyl)-1,3-dimethylpurine-2,6-dione	438	7.84	433	7.58	0.68
**77**	2-Hydroxy-2-phenylacetic acid	405	6.18	393	5.86	0.72
**78**	2-[(3-amino-2,4,6-triiodophenyl)methyl]butanoic acid	439	6.62	427	6.21	0.77
**79**	N-(2,6-Dimethylphenyl)-1-piperidine-2-carboxamide	403	5.53	385	5.78	0.78
**80**	(1S,3E,4R)-3-Benzylidene-1,7,7-trimethylbicyclo[2.2.1]heptan-2-one	371	5.50	351	5.60	0.79
**81**	2-Methylamino-1-phenylpropan-1-ol; hydrochloride	456	5.24	439	5.45	0.80

**82**	5-[[2-(3,4-Dimethoxyphenyl) ethyl](methyl)amino]-2-isopropyl-2-(3,4,5-trimethoxyphenyl)pentanenitrile	434	12.27	427	13.00	0.81
**83**	beta-3-Hydroxy-3-phenylbutanoic acid	357	4.70	330	5.40	0.81
**84**	1,2-Dibromoacenaphtene/5,6-dibromoacenaphthene	416	6.00	397	6.30	0.82
**85**	N-[[1-Ethylpyrrolidin-2-yl]methyl]-2-methoxy-5-sulfamoylbenzamide	460	11.03	451	10.04	0.83
**86**	3-(4-Fluorophenyl)-3-hydroxy- propanoic acid	381	6.60	362	7.40	0.83
**87**	Dimethyl O,O-diacetyl tartrate	378	6.60	358	6.50	0.84
**88**	2-(3-Fluorophenyl)-2-hydroxyacetic acid	394	5.46	369	5.56	0.85
**89**	N-(2,6-Dimethylphenyl)-1-propylpiperidine-2-carboxamide	414	5.69	391	10.64	0.85
**90**	2-(4-Fluorophenyl)-2-hydroxyacetic acid	426	7.08	406	6.90	0.89
**91**	1,5-Dichloro-11,12 dihydroxymethyl-9,10 dihydro-9,10 ethanoanthracene trans (exo)	527	12.80	520	12.80	0.90
**92**	3-(m-Chlorophenyl)-3-hydroxy propanoic acid	368	5.70	340	6.70	0.90
**93**	2-(2-Chlorophenyl)-2-hydroxy acetic acid	380	4.76	346	4.94	0.91
**94**	5-(2-Hydroxypropan-2-yl)-2-methylcyclohex-2-en-1-ol	424	8.22	405	8.29	0.92
**95**	2-Acetamido-N-methylacetamide	455	3.80	412	5.65	0.93
**96**	2-(2-Chlorophenyl)-2-hydroxy acetic acid	394	5.70	364	5.97	0.93
**97**	2-[4-(3-Oxo-1H-isoindol-2-yl)phenyl]butanoic acid	472	9.42	455	7.98	0.96
**98**	11,12-Di(iodomethyl)-9,10-dihydro-9,10-ethanoanthracene	491	8.00	469	8.40	1.01
**99**	2-(1-Nitronaphthalen-2-yl)-oxypropanamide	462	7.00	431	7.30	1.06
**100**	1-Naphthalen-1-yloxy-3-(propan-2-ylamino)- propan-2-ol; hydrochloride	468	7.13	436	7.96	1.09
**101**	2-(Methylamino)-1-phenylpropan-1-ol; hydrochloride	492	8.35	464	7.58	1.12
**102**	2-(1-Naphtoxy)propionamide	475	9.00	445	9.10	1.18
**103**	2-Acetamido-3-methylbutanoic acid	531	8.90	497	8.14	1.26
**104**	1-(1-Phenylethyl)thiourea	472	7.90	411	8.60	1.59

5b. Fitting of the gamma distribution

The CDF of the literature data were fitted to a cumulative gamma-distribution (Figure S6). The gamma-distribution was first corrected for the physical reality that ΔG^{Φ} protrudes in the negative domain. Therefore, we establish the adapted gamma-distribution γ (eq. 2):

$$\gamma\left(\Delta G^{\Phi}, \mathrm{k}, \theta\right) = \frac{1}{\theta^{\mathrm{k}}\,\Gamma(\mathrm{k})}\left(\Delta G^{\Phi}+\delta\right)^{\mathrm{k}-1} \mathrm{e}^{-(\Delta G^{\Phi}+\delta)/\theta} \quad \text{where } \Delta G^{\Phi}+\delta > 0 \qquad \text{(eq. 2)}$$

wherein γ is the probability density, Γ is the gamma function, *k* and *θ* serve as fitting parameters and *δ* provides the introduced offset. The cumulative gamma-distribution (CDF) then is calculated as eq. 3:

$$\mathrm{CDF}(\Delta G^{\Phi}, \mathrm{k}, \theta) = \int_{-\delta}^{\Delta G^{\Phi}} \gamma(G, \mathrm{k}, \theta)\, \mathrm{dG} \qquad \text{(eq. 3)}$$

We subsequently fitted the cumulative gamma-distribution (CDF, eq. 3) to the literature data by minimizing the sum of errors (least squares) through GRG nonlinear gradient descent. This provided the final fit shown in Figure S6 for which we found k = 3.171, θ = 0.284, δ = 0.459.

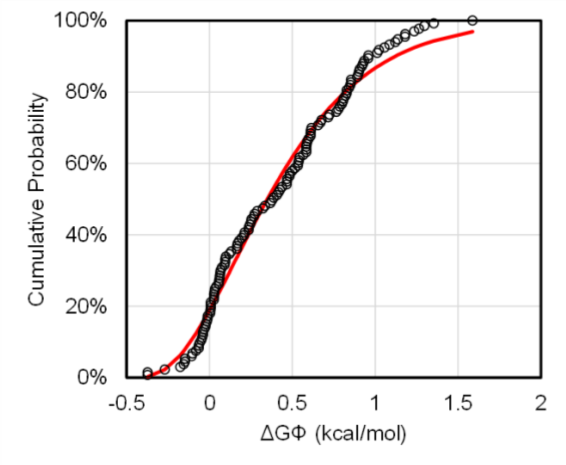

Figure S6: The cumulative probability distribution of the literature dataset of ΔG^{Φ} (black circles) fitted with an adapted gamma-distribution (eq. 2) through a GRG nonlinear gradient descent algorithm (red line).

5c. Statistical tests

First, a Goodness-of-Fit analysis (Chi-Square) was performed to assess whether the gamma-distribution accurately fitted the CDF of the combined dataset. The calculated value of $\chi^2 =$ 55.03283 under 132 degrees of freedom led to a p-value < α = 0.01 (***). Therefore, we have established that the fit is good.

Second, two-sided Kolmogorov-Smirnov tests were performed to assess whether the data from the individual ΔG^{Φ}-distributions (library **1**, library **2**, literature dataset) could indeed be inferred to originate from the fitted gamma distribution. Here, we found: D = 0.18746 and a p-value of 0.5283 for library **1**, D = 0.1783 and a p-value = 0.2469 for library **2**, and D = 0.062456 and a p-value = 0.6773 for the literature dataset. Under a typical α of 0.01, these results significantly accept the null-hypothesis, to mean that, indeed, all three individuals ΔG^{Φ}-distributions can be inferred to match the fitted gamma distribution and could originate from this mother distribution.

References

(1) Jacques, J.; Collet, A.; Wilen, S. H. *Enantiomers, Racemates, and Resolutions*; Krieger Publishing Company: Malabar, 1994.
(2) Noorduin, W. L.; Izumi, T.; Millemaggi, A.; Leeman, M.; Meekes, H.; Van Enckevort, W. J. P.; Kellogg, R. M.; Kaptein, B.; Vlieg, E.; Blackmond, D. G. Emergence of a Single Solid Chiral State from a Nearly Racemic Amino Acid Derivative. *J. Am. Chem. Soc.* **2008**, *130* (4), 1158–1159. https://doi.org/10.1021/ja7106349.
(3) Valenti, G.; Tinnemans, P.; Baglai, I.; Noorduin, W. L.; Kaptein, B.; Leeman, M.; ter Horst, J. H.; Kellogg, R. M. Combining Incompatible Processes for Deracemization of a Praziquantel Derivative under Flow Conditions. *Angew. Chem.* **2021**, *133* (10), 5339–5342. https://doi.org/10.1002/ange.202013502.

(4) Pinètre, C.; Ritou, L.; Gerard, C. J. J.; Cercel, H.; Leeman, M.; Kellogg, R. M.; Tinnemans, P.; Sanselme, M.; Brandel, C.; Dupray, V.; ter Horst, J. H. Rare Case of Polymorphism in the Binary System of Enantiomers of a Praziquantel Derivative. *Org. Process Res. Dev.* **2024**. https://doi.org/10.1021/acs.oprd.4c00035.
(5) Li, Z. J.; Zell, M. T.; Munson, E. J.; Grant, D. J. W. Characterization of Racemic Species of Chiral Drugs Using Thermal Analysis, Thermodynamic Calculation, and Structural Studies. *Journal of Pharmaceutical Sciences* **1999**, *88* (3), 337–346. https://doi.org/10.1021/js980205u.
(6) Charpentier, M. D. Crystallization in Multicomponent Chiral Systems: Thermodynamic Characterization and Guidelines for Chiral Resolution of Racemic Compounds with Cocrystallization, University of Strathclyde, 2023. https://stax.strath.ac.uk/concern/theses/6q182k65j.
